# Supplementary material for: PRMT5 Identified as a Viable Target for Combination Therapy in Preclinical Models of Pancreatic Cancer
Source: Biomolecules. 2025 Jun 30;15(7):948. doi: 10.3390/biom15070948 (PMC12292163; doi:10.3390/biom15070948)
Supplement: Supplementary file 1 [file biomolecules-15-00948-s001.zip › biomolecules-3681188-supplementary.pdf]

biomolecules

Article

PRMT5 identified as a viable target for combination therapy in preclinical models of pancreatic cancer

Xiaolong Wei[1,†], William J. Kane[1,†], Sara J. Adair[1], Sarbajeet Nagdas[2], Denis Liu[1], Todd W. Bauer[1,*]

Supplementary Materials

Figure S1. Effect of Gem and Ptx treatment on pancreatic tumor growth in orthotopic T.I. model. Human PDX 366 tumors were implanted orthotopically and tumor growth was monitored using MRI. **A.** Mouse weight change over time, relative to treatment start weight. **B.** Tumor volume as measured by MRI over time, relative to treatment start (24 days post-implantation). **C.** Absolute tumor volume as measured by MRI at 28 days of treatment (52 days post-implantation). **D.** Tumor weights at necropsy (56 days post-implantation, 32 days of treatment; Note: 4 mice not euthanized on this date to allow for tumor passage). Data presented as mean (SEM) for **A** and **B**, and mean (SD) for **C** and **D**. Gem=gemcitabine, Ptx=paclitaxel; 5 = 5 mg/kg I.P., 10 = 10 mg/kg I.P., 25 = 25 mg/kg I.P.; n=10 mice per group, *$P < 0.05$, **$P < 0.01$, ***$P < 0.001$, ****$P < 0.0001$.

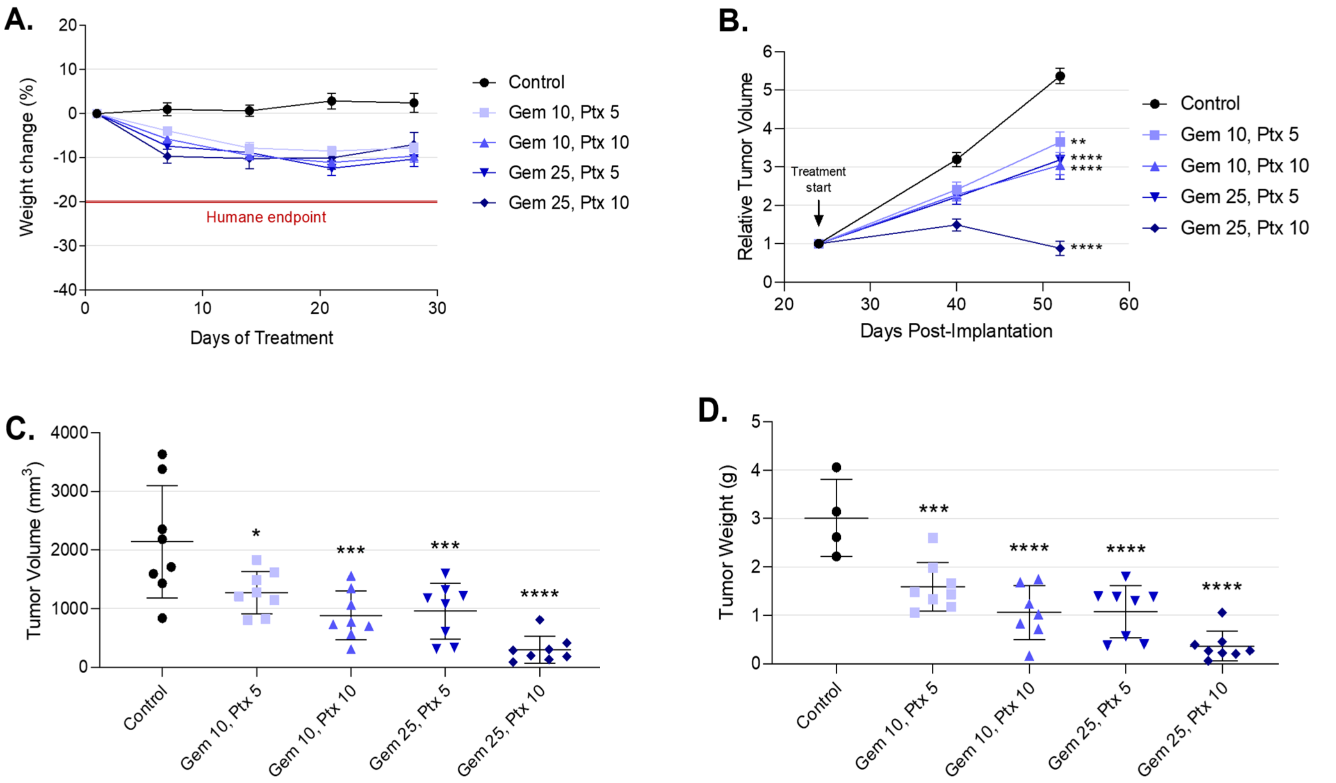

 https://doi.org/10.3390/biom15070948
